# Intraoperative Quantitative Measurements for Bradykinesia Evaluation during Deep Brain Stimulation Surgery Using Leap Motion Controller: A Pilot Study

**DOI:** 10.1155/2021/6639762

**Published:** 2021-06-15

**Authors:** Jingchao Wu, Ningbo Yu, Yang Yu, Haitao Li, Fan Wu, Yuchen Yang, Jianeng Lin, Jianda Han, Siquan Liang

**Affiliations:** ^1^Department of Neurosurgery, Tianjin Huanhu Hospital, Tianjin 300350, China; ^2^College of Artificial Intelligence, Nankai University, Tianjin 300350, China; ^3^Tianjin Key Laboratory of Intelligent Robotics, Nankai University, Tianjin 300350, China; ^4^Department of Neurological Rehabilitation, Tianjin Huanhu Hospital, Tianjin 300350, China

## Abstract

Deep brain stimulation (DBS) has shown a remarkably high effectiveness for Parkinson's disease (PD). In many PD patients during DBS surgery, the therapeutic effects of the stimulation test are estimated by assessing changes in bradykinesia as the stimulation voltage is increased. In this study, we evaluated the potential of the leap motion controller (LMC) to quantify the motor component of bradykinesia in PD during DBS surgery, as this could make the intraoperative assessment of bradykinesia more accurate. Seven participants with idiopathic PD receiving chronic bilateral subthalamic nucleus deep brain stimulation (DBS) therapy were recruited. The motor tasks of finger tapping (FT), hand opening and closing (OC), and hand pronation and supination (PS) were selected pre- and intraoperatively in accordance with the Movement Disorder Society revision of the Unified Parkinson's Disease Rating Scale. During the test, participants performed these tasks in sequence while being simultaneously monitored by the LMC and two professional clinicians. Key kinematic parameters differed between the preoperative and intraoperative conditions. We suggest that the average velocity ($\bar{V}$) and average amplitude ($\bar{A}$) of PS isolate the bradykinetic feature from that movement to provide a measure of the intraoperative state of the motor system. The LMC achieved promising results in evaluating PD patients' hand and finger bradykinesia during DBS surgery.

## 1. Introduction

Parkinson's disease (PD) is one of the most common neurodegenerative disorders, with an estimated prevalence of 1% in adults over 60 years old [1]. Deep brain stimulation (DBS) has been shown to be a highly effective treatment for PD. DBS is a surgery in which electrodes are implanted into deep brain structures; electrical signals generated by a neurostimulator that is implanted in the chest region below the collarbone can then be used to stimulate these structures. There have been over 100,000 patients implanted with DBS around the world in the past three decades [2].

Bradykinesia is the primary motor manifestation of PD and is usually a key disabling symptom, along with tremor and rigidity [3]. For many PD patients during DBS surgery, the therapeutic effects of the stimulation test are evaluated by assessing changes in bradykinesia as the stimulation voltage is increased. Different evaluators assess bradykinesia during operation in different ways but use similar principles: the baseline bradykinesia is assessed before any stimulation, and the changes in bradykinesia are evaluated by comparison with the baseline value using clinical scales such as the Unified Parkinson's Disease Rating Scale supported by the Movement Disorder Society (MDS-UPDRS) [4]. The MDS-UPDRS, which is scored based on a combination of multiple factors, such as movement speed, movement amplitude, and movement regularity, is the most widely used tool for assessing bradykinesia [5]. Intraoperative bradykinesia testing of PD patients is usually assessed by using the MDS-UPDRS (items 3.4–3.6). The majority of previous studies have suggested that such rating scales have low reliability and are highly dependent on the experience of the assessing neurologists [6]. Meanwhile, the clinical scales have only discrete levels and cannot be used for consecutive bradykinesia assessment. They may also be difficult to use intraoperatively and may be a source of bias. These limitations could be overcome if changes in bradykinesia were quantitatively measured and evaluated. Vaillancourt et al. [7] have shown that quantifying the components of exercise separately can make the evaluation more accurate.

There are many methods for quantifying PD motor symptoms using wearable motion sensors [8], potentiometers [9], gyrosensors [10], etc. Their main drawback is that they need to be attached to the fingers or hand, which may influence motor performance. Every gram of additional mass of the sensor will reduce the finger tremor by 0.85 Hz and also influence the acceleration amplitude [11]. In contrast to those devices, the leap motion controller (LMC) does not require placement of any sensors or markers on the body and thus has no effect on hand movements. This equipment can provide reliable measurements and has good consistency with marker-based motion capture systems [12].

In this study, we assessed the potential of the LMC to quantify the motor component of bradykinesia in PD during DBS surgery, as such quantification could make the intraoperative assessment of bradykinesia more accurate.

## 2. Materials and Methods

### 2.1. Participants

Seven participants (aged 61.9 ± 11.1 years, 4 males, 3 females) with idiopathic PD (mean disease duration: 9.3 ± 5.6 years, Hoehn and Yahr scores of 3 or 4) receiving chronic bilateral subthalamic nucleus deep brain stimulation (DBS) therapy were recruited. All patients fulfilled the inclusion criteria for DBS having a functional disability in daily life due to severe motor fluctuations or disabling tremor with a significant improvement of MDS-UPDRS in standardized L-dopa challenge. They were without surgical contraindications, dementia, or major ongoing psychiatric illness. Each patient provided informed consent. The study was approved by the Human Research Ethics Committee of Tianjin Huanhu Hospital (2020–53).

### 2.2. Leap Motion Controller

LMC is a consumer-grade sensor which is designed for hand gesture and finger position detection with high accuracy [13]. LMC utilizes miniature infrared cameras and inbuilt image-recognition algorithms to identify and estimate hand positions in three-dimensional space. The information regarding the user's hand, fingers, and gestures is captured between 2.5 and 60 cm above the center of the sensor. Since all three selected assessment tasks are based on hands' movement, LMC was selected as the sensor of the proposed assessment system. The feasibility of LMC in quantifying motor parameters for bradykinesia of PD patients has been explored [14]. Due to its small size (80 mm × 30 mm × 11.25 mm), light weight (45.4 g), and accurate positional measurement to within 0.01 mm [15], we hypothesized that it would work well in extracting data in a restrictive surgical environment.

### 2.3. Surgical Procedures

All patients underwent awake subthalamic nucleus (STN) DBS (Medtronic 3389 model, Medtronic Inc., Minneapolis, MN, USA) using Leksell frame. In order to design a method for intraoperative use, it is necessary to understand the DBS surgical procedure, which can be summed up as follows: (1) the anatomical target and the best path were planned using targeting software (Framelink 5.1, Medtronic, Minneapolis, MN) utilizing the computationally merged images from preoperative MRI and CT, avoiding sensitive structures (blood vessels, cerebral ventricles) during presurgical planning. (2) During the operation, two exploratory electrodes were inserted along the planned trajectories, and the target area was electrophysiologically mapped using microelectrode recording. (3) Intraoperative stimulation was performed from the microelectrode. After completion of test stimulation for all the positions, the permanent leads were finally implanted at the location with the best effect on the symptoms and the least side effects.

### 2.4. Intraoperative Assessment

The remaining stimulation parameters were kept constant with a voltage of 2 V, a pulse width of 60 *μ*s, and a frequency of 130 Hz using the permanent leads. Clinical evaluation of the best response was performed with the simultaneous use of subjective methods (MDS-UPDRS) and the LMC. The LMC was put in front of the participant and held by a bracket. Finger tapping (FT), opening and closing (OC), and hand pronation and supination (PS) motor tasks in line with the MDS-UPDRS were used. During the test, participants performed these tasks in sequence while being simultaneously monitored by the LMC and two professional clinicians. Each task was repeated and captured at least 10 times. The average clinical score for each task (the score of 0–4) was used as an indicator of the overall bradykinesia severity.

### 2.5. Data Processing

**Figure 1:**
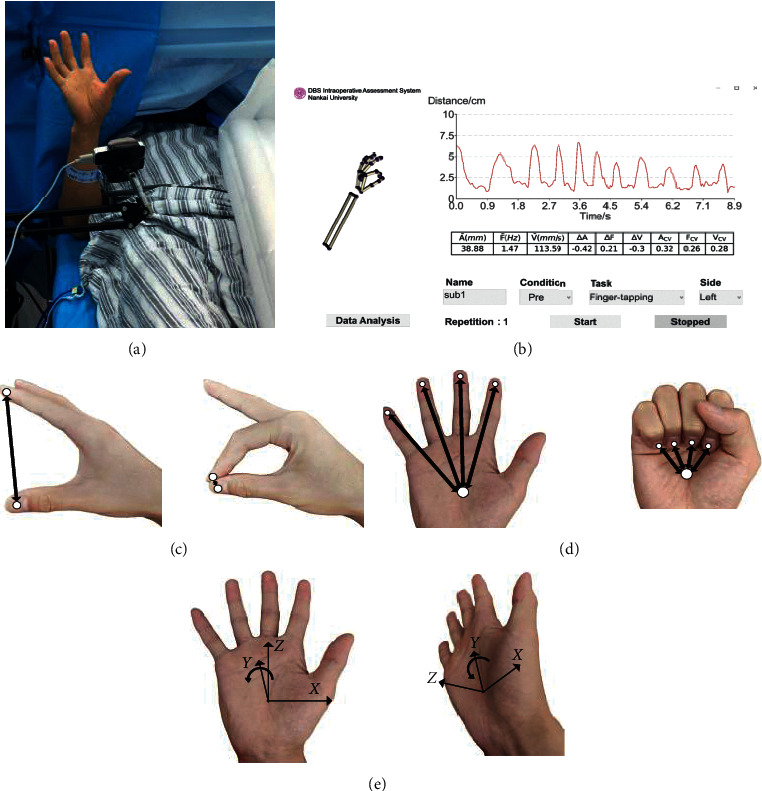
The software provides the real-time hand model and the trajectory of the movements to visualize the assessment process (a, b). Time-series kinematics extracted from the LMC for quantifying hand exercises included (c) FT: distance (mm) between distal joints of the thumb and index finger; (d) OC: average distance (mm) between the distal joints of the four fingers (except thumb) and the center of the palm; and (e) PS: roll angle (°) of the palm gesture which describes the rotational motion around the *y*-axis of the palm plane.

**Figure 2:**
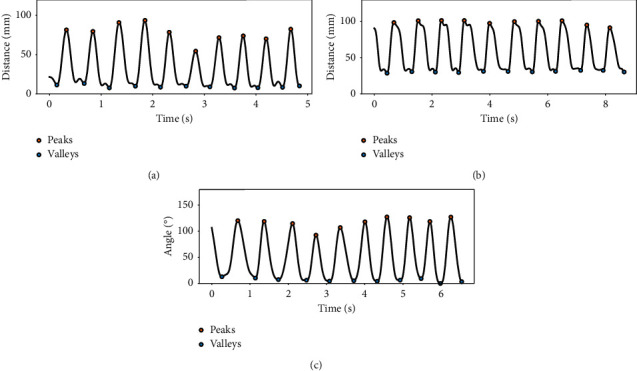
Representative segment of the kinematic signal reconstructed from (a) FT, (b) OC, and (c) PS. The black curve represents the 5 Hz low-pass filtered signal, the yellow dot represents the maximum value, and the blue dot represents the minimum value.

The motor parameters we used in this study are designed based on the MDS-UPDRS, and they reflect basic physical characteristics of the movements. Some studies have compared these parameters between PD patients and control groups and suggested significant difference between them [16]. The LMC provides position and orientation data from hand joints in time series. To record the raw data in the operating room, a graphical user interface (GUI) was developed using LMC's SDK (Software Development Kit, Ultraleap, Mountain View, CA, US). The GUI was developed in Unity 2019.4.4f1 version (Figures 1(a) and 1(b)). The data were processed to obtain kinematic signals describing each task (Figures 1(c)–1(e)). In order to eliminate high-frequency noise and the tremor component of PD patients' movement, a 5 Hz fourth-order Butterworth low-pass filter was implemented (Figure 2(a)–2(c)).

Patients' performances were quantified by frequency, amplitude, velocity, and rhythm. During each trial, 3 basic parameters were calculated for the first 10 movement cycles, respectively: (1) *F*: frequency; (2) *A*: amplitude, which was calculated by peak-to-peak value; (3) *V*: mean velocity. Then, 10 cycles' parameters were averaged to get $\bar{F}$, $\bar{A}$, and $\bar{V}$, respectively. Decrement ratios of frequency (Δ*F*), amplitude (Δ*A*), and velocity (Δ*V*) were obtained over the time course of the trial. Coefficients of variation *F*_*CV*_, *A*_*CV*_, and *V*_*CV*_ were calculated to quantify the variability of movement. Take the amplitude (*A*) as an example, let ${\bar{A}}_{1∼5}$ and ${\bar{A}}_{6∼10}$ be the mean amplitude of the first and last five movement cycles, respectively, and the decrement ratio of amplitude (Δ*A*) can be calculated as

(1) $$\begin{array}{c} \Delta A=\frac{{\bar{A}}_{6∼10}-{\bar{A}}_{1∼5}}{{\bar{A}}_{1∼5}}. \end{array}$$

Let *A*_std_ be the standard deviation of amplitude; the coefficient of variation of amplitude (*A*_*CV*_) can be calculated as

(2) $$\begin{array}{c} A_{CV}=\frac{A_{\text{std}}}{\bar{A}}. \end{array}$$

### 2.6. Statistical Analysis

Statistical analysis was performed using IBM SPSS Statistics 23.0. The different tasks were analyzed separately. Features in the preoperative and intraoperative conditions were analyzed as follows: Shapiro–Wilk tests were used to assess whether differences in extracted features between patients pre- and intraoperatively were normally distributed; significance was set at *P* > 0.05. Paired *t* tests (for normally distributed variables) or Wilcoxon tests (for other variables) were used to check if the difference was significant; significance was set at *P* < 0.05. Kendall's tau test was used to examine correlations between the extracted features and the corresponding MDS-UPDRS score given by two neurologists; significance was set at *P* < 0.05.

## 3. Results

A total of 84 trials were recorded (7 subjects × 2 hands × 2 conditions × 3 tasks). Each trial comprised two independent MDS-UPDRS clinical ratings matched to the corresponding LMC data. Missing data were apparent in 12 trials with the LMC.

The patients intraoperatively performed the FT task with significantly improved $\bar{V}$ and $\bar{A}$ compared with the patients preoperatively. Intraoperative lower scores on the MDS-UPDRS item for FT were correlated with improved $\bar{V}$, $\bar{A}$, and Δ*A* (Table 1).

The patients performed the OC task intraoperatively with significantly improved Δ*A* and *A*_*CV*_ compared with the patients preoperatively. Lower intraoperative scores on the MDS-UPDRS item for OC were correlated with improved Δ*V*and Δ*A* (Table 2).

The patients performed the PS task intraoperatively with significantly improved $\bar{V}$, $\bar{A}$, and Δ*A* compared with the patients preoperatively. Lower intraoperative scores on the MDS-UPDRS item for PS were correlated with improved $\bar{V}$ and $\bar{A}$, and lower *A*_*CV*_ (Table 3).

## 4. Discussion

In this pilot study, we developed a novel method for the preoperative and intraoperative bradykinesia evaluation in DBS surgery. The purpose was to test the hypothesis that changes in the patient's bradykinesia can be evaluated by use of a noninvasive, objective, simple, and sensitive method during DBS surgery. There are few reports of quantitative bradykinetic assessment during DBS surgery. To our knowledge, Papapetropoulos et al. [17] have used a mechanical setup to measure parameters of bradykinesia using the commercially available CATSYS system, while the patient underwent surgery. Compared with our method, it also enables bradykinesia measurement but is limited to evaluating finger-tapping frequency based on a simple touch-recording plate. Our assessment system was specifically designed for hand and finger movement including FT, OC, and PS in routine evaluation. Patients were asked to perform hand bradykinesia tasks traditionally evaluated during electrode implantation, thus without impeding or prolonging the surgical time. With the aid of the LMC, the improvement in bradykinesia can be measured quantitatively, and any assessments performed during DBS surgery can be reviewed and visualized.

It can be clearly seen from the results of these 7 patients that LMC is very sensitive to the measurement of changes in bradykinesia. The FT, OC, and PS were unaffected by changes in tremor amplitude because tremor oscillation signals were filtered out. The devices for quantitative evaluating the motor bradykinesia components of the hand and fingers showed promise with regard to FT, PS, and OC, key kinematic parameters that differed pre- and intraoperatively. Meanwhile, the quantitative results were correlated with the MDS-UPDRS scores, indicating that patients' motor characteristic features were successfully captured by the objective parameters. Unlike the findings of Lee et al. [5], both $\bar{V}$ and $\bar{A}$ appeared to be highly important in quantifying the FT task in our study yet showed poor correlation with the MDS-UPDRS ratings. Likewise, Δ*A* alone was best for assessing the OC task.

In our PS exercise, significant differences in $\bar{V}$ and $\bar{A}$ suggested that they are useful in distinguishing preoperative from intraoperative bradykinesia. Moreover, we calculated significant correlation coefficients between the MDS-UPDRS score and $\bar{V}$ (*r* = −0.403; *P* < 0.05), and MDS-UPDRS score and $\bar{A}$ (*r* = −0.480; *P* < 0.05) of PS. This indicates that there is a statistically significant relationship between the MDS-UPDRS score and the $\bar{V}$ and $\bar{A}$ of PS. The moderate correlations between the clinical scores and kinematic parameters were not surprising, because the hand-motion kinematic parameters may not represent the complete subject of a clinical evaluation of bradykinesia, which includes bradykinesia, hypokinesia, and motor coordination. This can be seen from the descriptions accompanying each MDS-UPDRS score [4]. In addition, it is not necessary to have strong agreement between quantitative indicators and subjective clinical scores for technological development [16]. Compared with the clinical scoring scales, technology-based measures should show higher sensitivity and reliability. PD is generally characterized by the gradual loss of speed and amplitude. We suggest that $\bar{V}$ and $\bar{A}$ of PS isolate the bradykinetic feature from that movement to provide a measure of the intraoperative state of the motor system.

Our study has certain limitations, including the small sample size and small battery of tests (rigidity and tremor were not measured). The method can objectively assess bradykinesia, but it has been applied only to postoperative analysis and not yet during the target selection procedure in the operating room. Thus, our next step will be visualizing these features and other information about the operation on the patient's anatomical images in real time so as to help the neurosurgeon during target selection.

## 5. Conclusions

The LMC achieved promising results in evaluating PD patients' hand and finger bradykinesia during DBS surgery. Separate quantification of $\bar{V}$ and $\bar{A}$ of PS is recommended for intraoperative testing. Future designs will incorporate the leap motion controller with a more user-friendly interface and real-time data processing.

## Figures and Tables

**Table 1:** Results of FT task statistical analysis.

FT features	Preoperative	Intraoperative	*P*-value	Correlation with MDS-UPDRS item
*Velocity* (*V*)				
** ** $\bar{V}$ (mm/s)	243.81 ± 93.43	348.48 ± 103.67	0.014^a^	−0.379^*∗*^
** **Δ*V*	−0.07 ± 0.23	−0.04 ± 0.14	0.709^a^	−0.240
** ** *V*_*CV*_	0.27 ± 0.08	0.23 ± 0.09	0.266^a^	−0.010

*Frequency* (*F*)				
** ** $\bar{F}$ (Hz)	1.74 (1.66, 2.39)	2.12 (1.71, 2.54)	0.286^b^	−0.202
** **Δ*F*	0.11 ± 0.13	0.05 ± 0.14	0.220^a^	0.238
** ** *F*_*CV*_	0.23 (0.13, 0.29)	0.17 (0.11, 0.20)	0.333^b^	0.216

*Amplitude* (*A*)				
** ** $\bar{A}$ (mm)	61.01 ± 18.64	80.15 ± 15.24	0.004^a^	−0.379^*∗*^
** **Δ*A*	−0.17 ± 0.22	−0.09 ± 0.11	0.362^a^	−0.353^*∗*^
** ** *A*_*CV*_	0.24 ± 0.09	0.18 ± 0.09	0.098^a^	0.151

FT, finger tapping; Δ, change; CV, coefficient of variation; PD, Parkinson's Disease; MDS-UPDRS-III, International Parkinson and Movement Disorders Society-sponsored revision of the Unified Parkinson's Disease Rating Scale. a: these *P* values were determined using paired *t* tests. b: these *P* values were determined using the Wilcoxon tests. Asterisks (*∗*) indicate significant correlations between kinematic parameters and the score on the corresponding MDS-UPDRS item: ^*∗*^*P* < 0.05; ^*∗∗*^*P* < 0.01.

**Table 2:** Results of OC task statistical analysis.

OC feature	Preoperative	Intraoperative	*P*-value	Correlation with MDS-UPDRS item
*Velocity* (*V*)				
** ** $\bar{V}$ (mm/s)	187.37 ± 43.02	208.51 ± 52.83	0.079^a^	−0.229
** **Δ*V*	−0.14 ± 0.23	−0.03 ± 0.14	0.104^a^	−0.356^*∗*^
** ** *V*_*CV*_	0.21 ± 0.12	0.20 ± 0.07	0.732^a^	0.085

*Frequency* (*F*)				
** ** $\bar{F}$ (Hz)	1.58 ± 0.39	1.67 ± 0.48	0.176^a^	−0.250
** **Δ*F*	0.01 ± 0.18	0.02 ± 0.1	0.829^a^	−0.133
** ** *F*_*CV*_	0.19 ± 0.11	0.20 ± 0.08	0.679^a^	−0.085

*Amplitude* (*A*)				
** ** $\bar{A}$ (mm)	58.04 ± 9.66	59.85 ± 6.53	0.521^a^	−0.124
** **Δ*A*	−0.07 (−0.19, 0.02)	−0.03 (−0.05, 0.01)	0.028^b^	−0.345^*∗*^
** ** *A*_*CV*_	0.15 ± 0.12	0.09 ± 0.07	0.034^a^	0.231

OC, hand opening/closing; Δ, change; CV, coefficient of variation; PD, Parkinson's Disease; MDS-UPDRS-III, International Parkinson's and Movement Disorders Society-sponsored revision of the Unified Parkinson's Disease Rating Scale. a: these *P* values were determined using paired *t* tests. b: these *P* values were determined using the Wilcoxon test. Asterisks (*∗*) indicate significant correlations between kinematic parameters and the score on the corresponding MDS-UPDRS item: ^*∗*^*P* < 0.05; ^*∗∗*^*P* < 0.01.

**Table 3:** Results of PS task statistical analysis.

PS features	Preoperative	Intraoperative	*P* value	Correlation with MDS-UPDRS item
*Velocity (V*)				
** ** $\bar{V}$ (roll angle,°)	357.95 ± 144.88	604.75 ± 205.24	0.000^a^	−0.403^*∗*^
** **Δ*V*	−0.11 ± 0.17	0.04 ± 0.25	0.183^a^	−0.109
** ** *V*_*CV*_	0.16 (0.13, 0.26)	0.17 (0.14, 0.21)	0.638^b^	0.180

*Frequency (F*)				
** ** $\bar{F}$ (Hz)	1.71 (1.44, 2.02)	1.85 (1.54, 2.40)	0.075^b^	0.066
** **Δ*F*	0.06 ± 0.17	0.05 ± 0.17	0.943^a^	−0.051
** ** *F*_*CV*_	0.19 ± 0.15	0.16 ± 0.12	0.680^a^	0.223

*Amplitude* (*A*)				
** ** $\bar{A}$ (°)	104.04 ± 37.53	151.95 ± 34.24	0.000^a^	−0.480^*∗∗*^
** **Δ*A*	−0.19 ± 0.14	−0.01 ± 0.12	0.006^a^	−0.168
** ** *A*_*CV*_	0.17 ± 0.09	0.13 ± 0.09	0.200^a^	0.316^*∗*^

PS, pronation-supination movements of hands; Δ, change; CV, coefficient of variation; PD, Parkinson's Disease; MDS-UPDRS-III, International Parkinson's and Movement Disorders Society-sponsored revision of the Unified Parkinson's Disease Rating Scale. a: these *P* values were determined using paired *t* tests. b: these *P* values were determined using the Wilcoxon test. Asterisks (*∗*) indicate significant correlations between kinematic parameters and the score on the corresponding MDS-UPDRS item: ^*∗*^*P* < 0.05; ^*∗∗*^*P* < 0.01.

## Data Availability

The data used to support the findings of this study are available from the corresponding author upon request.

## References

[1] Strauss I., Kalia S. K., Lozano A. M. (2014). Where are we with surgical therapies for Parkinson’s disease?. *Parkinsonism and Related Disorders*.

[2] Shah A., Coste J., Lemaire J.-J. (2017). A novel assistive method for rigidity evaluation during deep brain stimulation surgery using acceleration sensors. *Journal of Neurosurgery*.

[3] Goldenberg M. M. (2008). Medical management of Parkinson’s disease. *P and T A Peer-Reviewed Journal for Formulary Management*.

[4] Goetz C. G., Tilley B. C., Shaftman S. R. (2008). Movement disorder society-sponsored revision of the unified Parkinson’s disease rating scale (MDS-UPDRS): scale presentation and clinimetric testing results. *Movement Disorders*.

[5] Lee W. L., Sinclair N. C., Jones M. (2019). Objective evaluation of bradykinesia in Parkinson’s disease using an inexpensive marker-less motion tracking system. *Physiological Measurement*.

[6] Daneault J.-F., Carignan B., Sadikot A. F., Duval C. (2013). Are quantitative and clinical measures of bradykinesia related in advanced Parkinson’s disease?. *Journal of Neuroscience Methods*.

[7] Vaillancourt D. E., Prodoehl J., Verhagen Metman L., Bakay R. A., Corcos D. M. (2004). Effects of deep brain stimulation and medication on bradykinesia and muscle activation in Parkinson’s disease. *Brain A Journal of Neurology*.

[8] Dai H., Lin H., Lueth T. C. (2015). Quantitative assessment of parkinsonian bradykinesia based on an inertial measurement unit. *BioMedical Engineering OnLine*.

[9] Oliveira R. M., Gurd J. M., Nixon P., Marshall J. C., Passingham R. E. (1998). Hypometria in Parkinson’s disease: automatic versus controlled processing. *Movement Disorders Official Journal of the Movement Disorder Society*.

[10] Kim J.-W., Lee J.-H., Kwon Y. (2011). Quantification of bradykinesia during clinical finger taps using a gyrosensor in patients with Parkinson’s disease. *Medical and Biological Engineering and Computing*.

[11] Fraiwan L., Khnouf R., Mashagbeh A. R. (2016). Parkinson’s disease hand tremor detection system for mobile application. *Journal of Medical Engineering and Technology*.

[12] Smeragliuolo A. H., Hill N. J., Disla L., Putrino D. (2016). Validation of the Leap Motion Controller using markered motion capture technology. *Journal of Biomechanics*.

[13] Weichert F., Bachmann D., Rudak B., Fisseler D. (2013). Analysis of the accuracy and robustness of the leap motion controller. *Sensors*.

[14] Kim M. J., Naydanova E., Hwang B. Y., Mills K. A., Anderson W. S., Salimpour Y. Quantification of Parkinson’s disease motor symptoms: a wireless motion sensing approach.

[15] Butt A. H., Rovini E., Dolciotti C. (2018). Objective and automatic classification of Parkinson disease with Leap Motion controller. *BioMedical Engineering OnLine*.

[16] Bank P. J. M., Marinus J., Meskers C. G. M., de Groot J. H., van Hilten J. J. (2017). Optical hand tracking: a novel technique for the assessment of bradykinesia in Parkinson’s disease. *Movement Disorders Clinical Practice*.

[17] Papapetropoulos S., Jagid J. R., Sengun C., Singer C., Gallo B. V. (2008). Objective monitoring of tremor and bradykinesia during DBS surgery for Parkinson disease. *Neurology*.

